# Superficial radial nerve–lateral antebrachial cutaneous nerve anatomic variation

**DOI:** 10.1002/brb3.195

**Published:** 2013-12-01

**Authors:** Eduardo R Davidovich, Osvaldo J M Nascimento

**Affiliations:** Neurology and Neuroscience Clinical Research Sub-Unit (Neuro-UPC) – Hospital Universitário Antonio Pedro, Universidade Federal Fluminense (UFF)Rio de Janeiro, Brazil

**Keywords:** Anatomic variation, hand dorsum, lateral antebrachial cutaneous nerve, nerve conduction, superficial radial nerve

## Abstract

**Introduction:**

This study focuses on an anatomic variation in which the lateral antebrachial cutaneous nerve (LACN) innervates the radial border of the dorsum of the hand and thumb in addition to, or replacing, the superficial radial nerve (RSN). Here, we propose a technique of nerve conduction that identifies this variation.

**Methods:**

We studied nerve conduction in 200 upper limbs of two series of 50 volunteers. We sought evidence of the aforementioned variation on the dorsum of the hand and in the thumb.

**Results:**

We found eight occurrences of this variation on the dorsum of the hand and 11 variants on the thumb within the two respective series of 100 upper limbs studied.

**Discussion:**

The RSN–LACN anatomic variation can be studied using nerve conduction. The knowledge of this variation is particularly important for the evaluation of proximal radial nerve injury.

## Introduction

The interpretation of nerve conduction and electromyography studies (EDX) in cases of focal lesions of peripheral nerves is based on detailed knowledge of the anatomy of nerves and muscles. While anatomic variations in motor nerves are well-documented in the literature and in textbooks of electromyography, variations in the sensory nerves, especially those innervating the dorsum of the hand, are less well-discussed (Oh 1993; Aminoff 1998; Kmura 2001; Dumitru et al. 2002). This is despite the variations in sensory innervation being relatively frequent in the patient population (Auerbach et al. 1994; Grossman et al. 1998; Bas and Kleinert 1999; Mok et al. 2006).

The idea for this study came from the observation of a patient with a complete lesion of the radial nerve (RN), in the arm segment and preservation of the superficial radial nerve (RSN) sensory nerve action potential (SNAP). This case could be explained by an anatomic variation (Davidovich et al. 2013). As anatomic variation may complicate the interpretation of nerve conduction data, we searched for a nerve conduction technique for evaluating this particular problem, but were unable to find any in the literature at that time. The focus of this study is to call attention to a poorly known anatomic variation, in which the lateral antebrachial cutaneous nerve (LACN) innervates the radial border of the dorsum of the hand in addition to, or replacing, the RSN. We propose a technique of nerve conduction to identify this variation.

Leis and Wells (2008) published an elegant nerve conduction study of the RSN and ulnar nerve anatomic variation on the dorsum of the hand; they demonstrated that nerve conduction studies can be useful tools for evaluating variations in sensory innervation of the dorsum of the hand. Leis et al. (2010) found an important clinical use for the technique described in 2008. To our knowledge, no nerve conduction technique to evaluate the occurrence of the RSN–LACN anatomic variation on the dorsum of the hand has been published.

## Methods

We studied 100 volunteers (200 upper limbs) of the EDX lab at Antonio Pedro University Hospital. None of the volunteers included in this study had clinical evidence of RN, RSN, or LACN dysfunction. All volunteers underwent a standard upper limb EDX, including sensory nerve conduction of the RSN, median, and ulnar nerves and motor nerve conduction of the median and ulnar nerves. The volunteers were divided into two series: in the A series (*n* = 50), we looked for anatomic variation in the dorsum of the hand; and in the B series (*n* = 50), we looked for anatomic variation in the first finger. The Institutional Review Board approved the clinical research and we obtained informed consent from all subjects.

We used a Medelec Synergy (Oxford Instrument, Surrey, U.K.) 2-channel EDX machine, with the range of upper and lower frequency filter of sensory nerve conduction set from 20 Hz to 2 kHz. In addition, sweep speed was maintained at 2 msec/division in channel 1 and at 1 msec/division in channel 2, with sensitivity at 20 *μ*V/division. Averaging techniques and increasing the gain of the screen were used to access small amplitude potentials. The stimulation duration was maintained at 0.1 msec, and the intensity was increased gradually until the maximal sensory response was achieved. When needed, skin temperature was increased with a portable heater to above 32°C. Latencies were measured to the peak of the negative deflection, and amplitudes were measured from baseline to the negative peak.

The nerve conduction technique used was a variation in the Spindler and Felsenthal technique for LACN nerve conduction (channel 2) (Spindler and Felsenthal 1978), and included a second channel (channel 1) for simultaneous capture of antidromic SNAP on the radial border of the dorsum of the hand in 50 patients (A series) or on the thumb in 50 patients (B series). The electric stimulus was applied lateral to the biceps tendon in the elbow where the LACN nerve pierces the superficial fascia and becomes a subcutaneous nerve.

The proximity of RN and LACN in the lateral border of the biceps tendon was an element of great concern, due to the possibility of costimulation. In the stimulus point, the LACN lies in the subcutaneous tissue. At this same point, the RN is located much deeper, below the superficial fascia and between the brachioradialis and brachialis muscles. The difference in depth of these two nerves is related to the current intensity necessary to stimulate each one. To stimulate RN it is necessary to use larger currents than is necessary to stimulate only the LACN.

To minimize the possibility of costimulation of RN, we use the minimum stimulus intensity required for the obtention of a clear LACN SNAP on channel 2. When RN was also stimulated, a motor artifact could be easily identified on channel 2. All patients in whom this artifact was identified were excluded from the study.

For the LACN SNAP (channel 2), a plastic-mounted bipolar electrode (surface disks 3 cm apart) was placed with the active recording electrode 10–12 cm distal to the biceps tendon in a line from the biceps tendon to the radial artery on the wrist. The reference electrode was placed distally.

**Figure 1:**
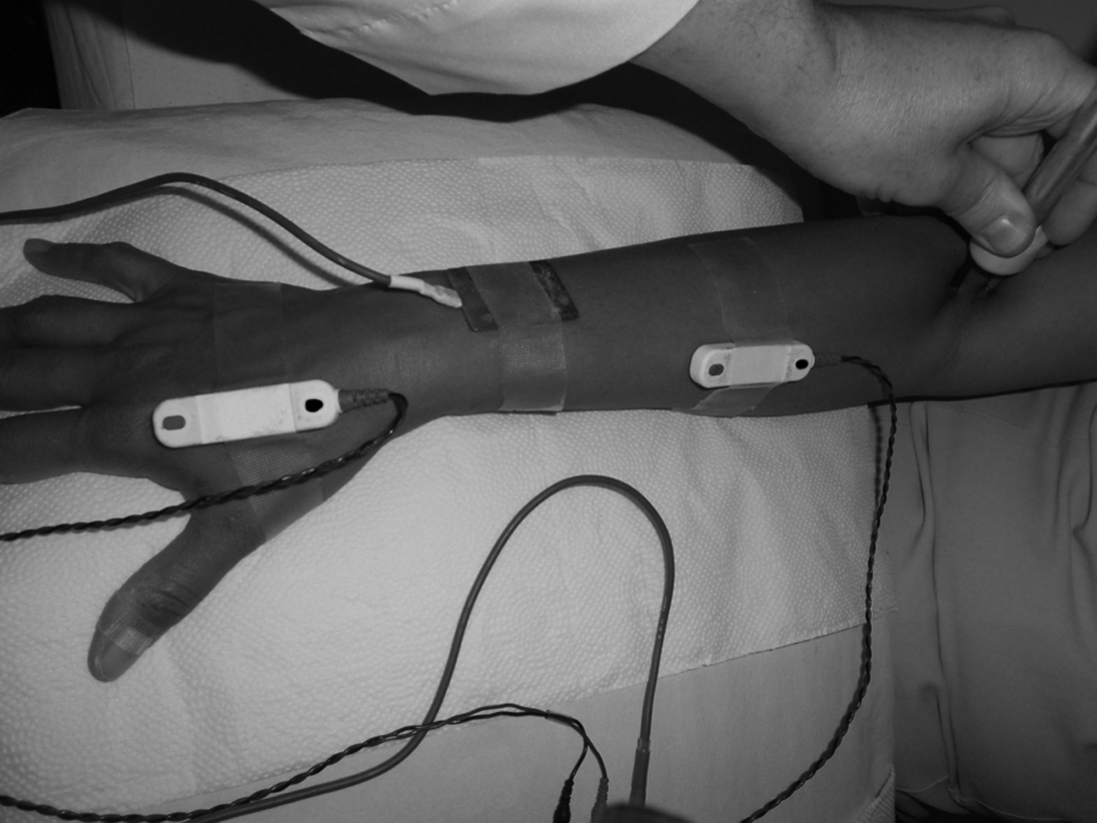
Assembly for the realization of the A series. The image was made with the forearm pronated for better visualization. The nerve conduction data were obtained with the forearm supinated.

**Figure 2:**
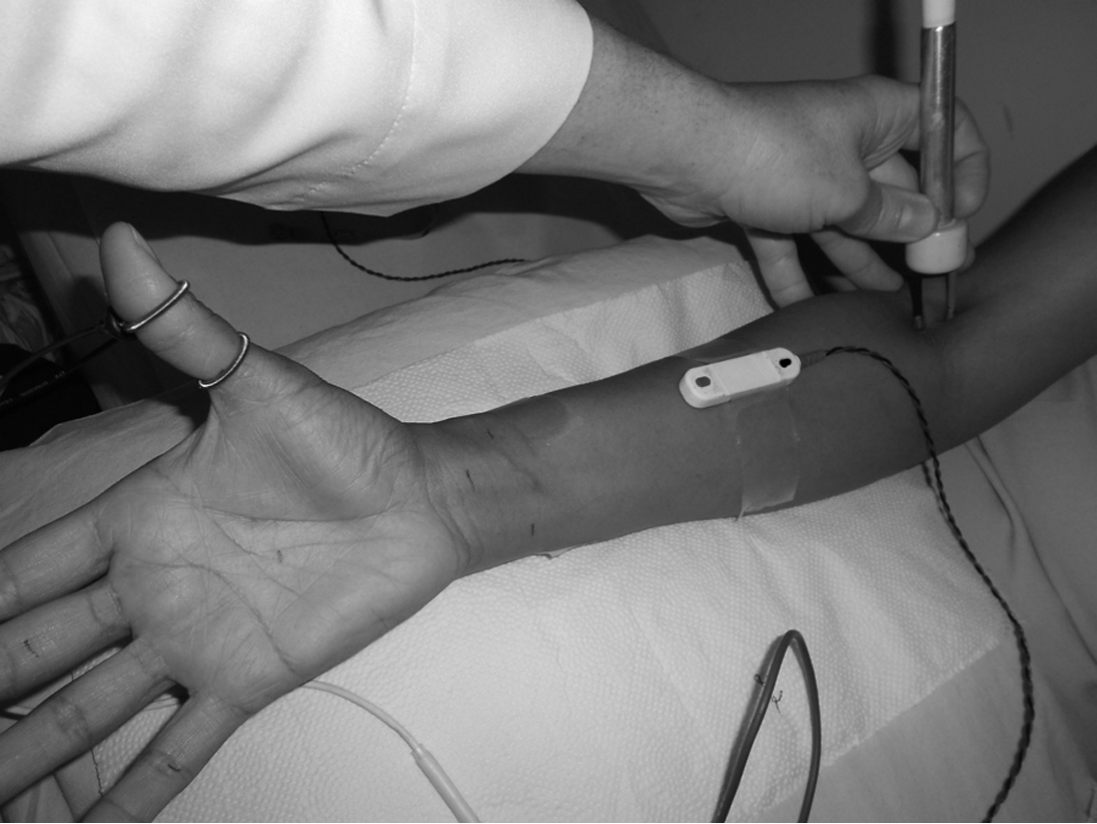
Assembly for the realization of the B series.

In the A series (Fig. 1), the active and reference electrodes for channel 1 were plastic-mounted bipolar electrodes (surface disks 3 cm apart); the active recording electrode was placed at the apex of the “V” between the first and second metacarpal bones, and the reference electrode was placed distally. In the B series (Fig. 2), the active and reference electrodes for channel 1 were rings mounted on the thumb, with the active electrode proximal and the reference electrode 3 cm distal.

**Figure 3:**
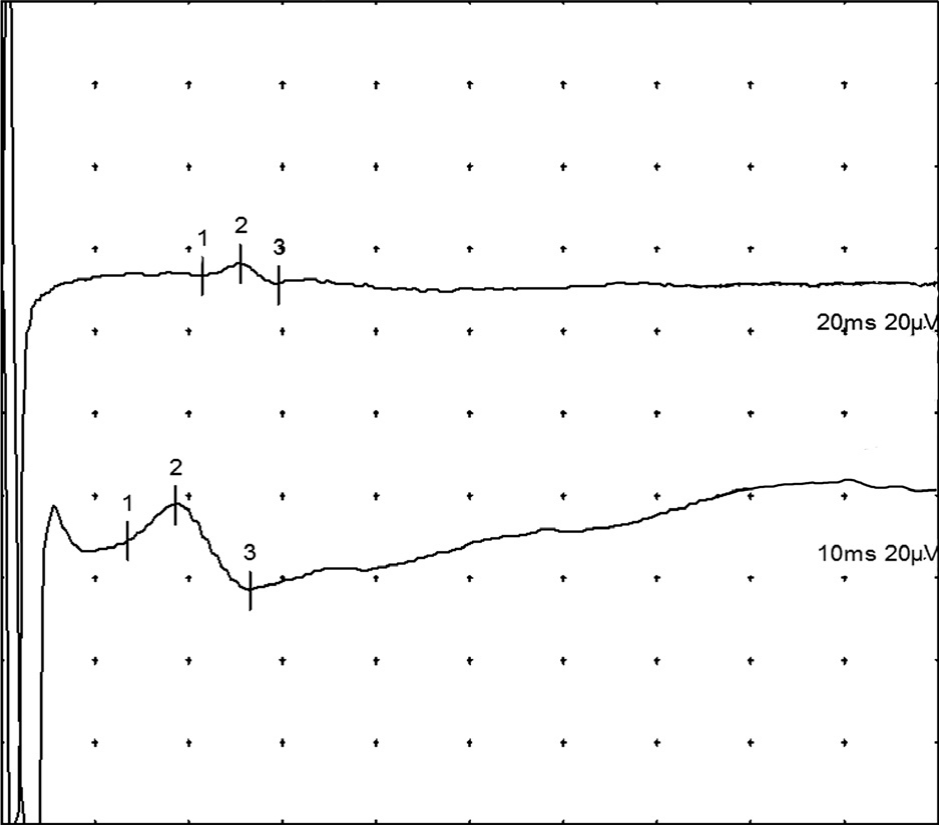
An example of an upper limb positive in the A series. The upper curve represents the channel 1, with the SNAP obtained in the dorsum of the hand. The lower curve represents the channel 2 with the LACN SNAP obtained by standard technique.

**Figure 4:**
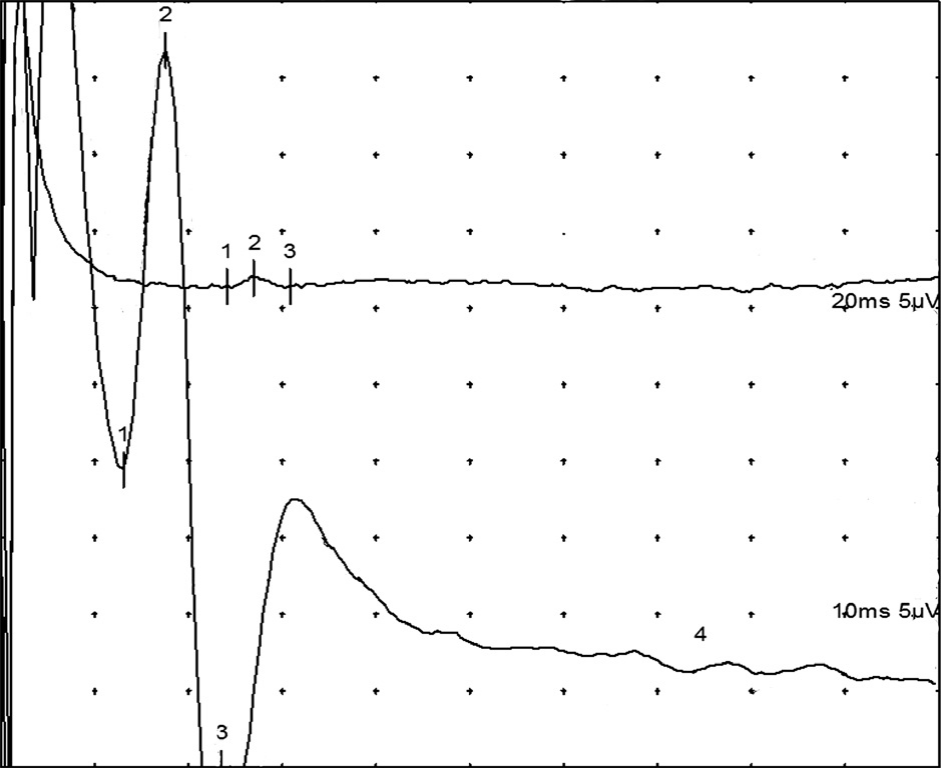
An example of an upper limb positive in the B series. The upper curve represents the channel 1, with the SNAP obtained in the first finger. The lower curve represents the channel 2 with the LACN SNAP obtained by standard technique.

We considered the measurement to be positive for variation in the upper limbs when a SNAP was obtained on channel 1, whereas channel 2 showed a clear LACN SNAP (Fig. 3, 4). We believe that the SNAP captured on channel 1, an area normally supplied by the RSN, originates from the variant LACN.

Even with the precautions taken in the stimulation and in the exclusion of patients with motor artifact, costimulation of RN remains as a possible pitfall of this technique.

## Results

Of the 50 patients in the A series, 10 were male (20%) and 40 female (80%). We found six patients (12%) who tested positive for the RSN–LACN anatomic variation; all of them were females. We found two patients (4%) who tested positive for the variation in both upper limbs. Of the 100 upper limbs studied, we observed eight (8%) that were positive for the variation. All the four patients with unilateral variation showed variation on the left side. The SNAP amplitude obtained in channel 1 in the eight positive limbs ranged from 0.74 to 10.6 *μ*V, with an average of 5.2 *μ*V.

Of the 50 patients in the B series, 10 were male (20%) and 40 female (80%). We found 11 patients (22%) who tested positive for the RSN–LACN anatomic variation; all of them were females. We did not find bilateral variation in the B series. Of the 100 upper limbs studied, we observed 11 (11%) that were positive for the variation. In 11 patients with unilateral variation, six (54.5%) showed the variation on the left side, and five (45.5%) had the variation on the right side. The SNAP amplitude obtained in channel 1 in the 11 positive limbs ranged from 0.70 to 6.2 *μ*V, with an average of 2.4 *μ*V.

## Discussion

Anatomic variations in peripheral nerves may promote misinterpretation of neurophysiological findings in clinical practice. Most are known only anatomically. As an example, RSN and LACN anatomic variations are described only in textbooks of anatomy, and dissecting cadaver study reports. To our knowledge, this is the first RSN–LACN anatomic variation nerve conduction study report.

Appelton (1911) first described this variation in the literature in a dissection of a forearm, where the RN below the elbow presented only the posterior interosseous branch, and the RSN was absent. On the dorsum of the hand, the LACN extended out beyond its usual distribution to supply the RSN territory. Another very interesting finding was the presence of branches of the dorsal ulnar nerve greater than those usually observed, completing the dorsal hand innervation. Since this initial report, several studies have observed this variation in cadavers and patients. Clinical examination findings in more than 1000 gunshot injuries of peripheral nerves cases were reported by Stopford (1918). There were 67 cases of proximal RN injury. Of these, two patients had no area of cutaneous anesthesia which may represent replacement of the innervation of the dorsum of the hand by the LACN. The area of anesthesia in other cases of this series showed great variability, which may correspond to distinct degrees of branching communication between the ulnar nerve, RSN, and LACN on the dorsum of the hand.

Mackinnon and Dellon (1985) studied the distribution of LACN and RSN by anatomic dissection of 53 cadavers and 41 surgical dissections. Of these, 75% had partial or total communication between the LACN nerve and RSN on the dorsum of the hand. Additionally, Madhavi and Holla (2003) reported a case of dual innervation of the dorsum of the thumb by the RSN and LACN in a cadaver dissection. Mok et al. (2006) studied the sensory innervation in 30 cadaver forearms. In this study, one in three forearms presented connections between RSN and LACN. In one case, the LACN was the major contributor to the dorsal thumb innervation. Also focusing on anatomic study, Huanmanop et al. (2007) did dissections of the RSN in 79 upper limbs of 40 Thai cadavers. In this study, the occurrence of communication between the LACN nerve and RSN was 43%. Furthermore, in 2.5% of the upper limbs, the RSN was replaced by the LACN. Yogesh et al. (2011) reported a case of cadaver dissection in which the RN and musculocutaneous nerves had unilateral anatomic variation. In this case, the RN ended after the branches to the triceps muscle. The musculocutaneous nerve was responsible for sensory innervation of the radial border of the dorsum of the hand and the motor innervation of the brachioradialis, extensor carpi radialis, and all the muscles supplied by the posterior interosseous nerve.

In this neurophysiological study, we found evidence of the RSN–LACN variation in the dorsum of the hand in six of 50 patients (12%), and two of them (4%) had bilateral variation. In 100 upper limbs, 8% had the variation. Of 50 patients whose thumbs were tested, we found evidence of this variation in 22% (1 in 4 patients). None of them had bilateral variation. In 100 upper limbs, 11% had the variation. As the RSN–LACN variation had never been studied by nerve conduction, our data cannot be directly compared with the literature. In our series of 200 upper limbs, we found RSN–LACN variation in 19 (9.5%) limbs. This prevalence, seen in our conduction study, is lower than that found by others, but still suggests that this variation is relatively frequent (Mackinnon and Dellon 1985; Mok et al. 2006; Huanmanop et al. 2007).

The frequency of variant members found in our study, compared with anatomical studies, seems to confirm that the RN costimulation is not a significant problem for our technique. The costimulation of the RN would tend to increase the frequency of variant members found.

Thus, anatomic studies have demonstrated a high occurrence of communication between the RSN and LACN. However, for the neurophysiologist, it is important to know how often this variation can interfere with the RSN SNAP during a neuroconduction study. With regard to this information, the data obtained in this study are unique. In the case of occurrence of the RSN–LACN variation, the EDX examination may be impaired when evaluating proximal lesions of the RN in the arm, lesions of the distal RSN in the wrist, and in LACN injuries. In particular, in cases of proximal RN injury, the occurrence of this variation may lead to diagnostic errors in the EDX, with a total axonal injury erroneously assessed as a partial lesion with a conduction block component. Eventually, this misinterpretation can delay the indication for surgical repair for complete nerve injuries, leading to worse prognosis for functional recovery.

A better knowledge of the anatomic variations in the peripheral nerves, and their manifestations in nerve conduction studies, has practical utility for EDX examination. Such knowledge helps better interpretation of the data obtained when these variations are present in normal cases or in pathological conditions.

We believe that the technique presented in this study can be useful when an examiner encounters a patient with evidence of complete RN lesion in the motor nerve conduction and needle examinations and showing preservation of the RSN SNAP.
